# Molecularly Imprinted Polymers for Pharmaceutical Impurities: Design and Synthesis Methods

**DOI:** 10.3390/polym15163401

**Published:** 2023-08-14

**Authors:** Aliya Nur Hasanah, Ike Susanti

**Affiliations:** 1 Pharmaceutical Analysis and Medicinal Chemistry Department, Faculty of Pharmacy, Universitas Padjadjaran, Jalan Raya Bandung Sumedang KM 21 Jatinangor, Bandung 45363, Indonesia; ike14001@mail.unpad.ac.id; 2 Drug Development Study Center, Faculty of Pharmacy, Universitas Padjadjaran, Jalan Raya Bandung Sumedang KM 21, Sumedang 45363, Indonesia

**Keywords:** impurity, molecularly imprinted polymers, pharmaceutical product

## Abstract

The safety of a medicinal product is determined by its pharmacological and toxicological profile, which depends not only on the active substance’s toxicological properties, but also on the impurities it contains. Because impurities are a problem that must be considered to ensure the safety of a drug product, many studies have been conducted regarding the separation or purification of active pharmaceutical ingredients (APIs) and the determination of impurities in APIs and drug products. Several studies have applied molecularly imprinted polymers (MIPs) to separate impurities in active ingredients and as adsorbents in the sample preparation process. This review presents the design of MIPs and the methods used to synthesise MIPs to separate impurities in APIs and drug product samples, the application of MIPs to separate impurities, and a view of future studies involving MIPs to remove impurities from pharmaceutical products. Based on a comparison of the bulk and surface-imprinting polymerisation methods, the MIPs produced by the surface-imprinting polymerisation method have a higher adsorption capacity and faster adsorption kinetics than the MIPs produced by the bulk polymerisation method. However, the application of MIPs in the analysis of APIs and drug products are currently only related to organic compounds. Considering the advantages of MIPs to separate impurities, MIPs for other impurities still need to be developed, including multi-template MIPs for simultaneous separation of multiple impurities.

## 1. Introduction

**Figure 1:**
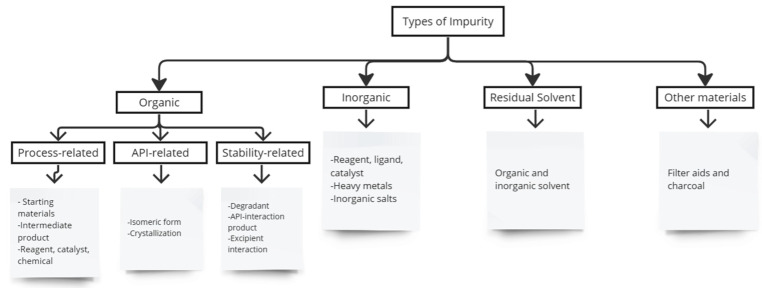
The types of impurities in pharmaceutical products.

The pharmaceutical industry aims to protect public health by ensuring that patients have access to the right medicine at the correct dose and potency and at an affordable price. Therefore, drug safety and efficacy are two major issues in drug therapy [1]. The safety of a medicinal product is determined by its pharmacological and toxicological profile, which depends not only on the active substance’s toxicological properties, but also on the impurities it contains [1]. Impurities are unwanted chemicals found in pharmaceuticals that occur during formulation or the manufacturing process, or arise from degradation of active pharmaceutical ingredients (APIs) and drug products. They are not chemicals that have been added intentionally [2,3]. According to the ICH, impurities are not active ingredients or excipients of drug products [4]. Impurities are classified as organic compounds, residual solvents, and inorganic impurities (Figure 1) [1,5]. Impurities can result from chemical changes in drug substances during drug product manufacturing and storage due to light, temperature, pH, water, and reactions with excipients [4].

Even in small amounts, impurities can reduce the safety and effectiveness of pharmaceutical products. Therefore, API impurity profiling is becoming increasingly important, as impurities in APIs can compromise drug safety and quality [6]. In 2018, *N*-nitrosodimethylamine (NDMA) was found in valsartan as an impurity. In 2022, Mansouri et al. [7] evaluated the risk of cancer associated with exposure to NDMA-contaminated valsartan. They found a slightly increased risk of liver cancer and melanoma in patients exposed to NDMA via routine valsartan treatment. NDMA is produced in the process of synthesising raw materials of the sartan group. The generation of this group requires *N*,*N*-dimethylformamide as a solvent and sodium nitrite as a reagent to form the tetrazole ring [7]. In 2022, Indonesia reported a significant increase in acute kidney injury (AKI) cases in children. On 5 February 2023, over 300 cases were reported, and over half resulted in death. The cases were associated with ethylene glycol (EG) and diethylene glycol (DEG) impurities in an oral solution product [8]. DEG and EG are impurities in raw excipient materials such as glycerine and propylene glycol [9,10]. Several types of impurities in APIs are considered to be genotoxic impurities (GTIs). These compounds can cause genetic mutations, chromosomal breaks, and/or chromosomal rearrangements, resulting in cancer [11]. Therefore, it is crucial to remove impurities in pharmaceutical products.

The detection of impurities in APIs and drug products is critical to ensure the safety of a product. In this endeavour, the preparation and analytical methods are essential to determine the impurity. Table 1 shows several preparation methods and instruments that have been used to analyse impurities in APIs and drug products.

Molecularly imprinted polymers (MIPs) have received great attention for their ability to selectively separate analytes in various samples. These synthetic polymers have a defined selectivity for a particular analyte or group of structurally related compounds, making them ideal materials for separation processes [20]. MIPs are used as an adsorbent for solid-phase extraction (SPE) [21,22], dispersive solid-phase extraction (D-SPE) [23,24], pipette solid-phase extraction (P-SPE) [25,26], and monolithic columns [27]. MIPs have also been applied to separate impurities from APIs or to purify APIs. The use of MIPs for impurity removal from APIs is quite suitable, considering that the impurity level is low and these MIPs can bind selectively and sensitively to template molecules (analytes). Until now, no review has discussed how to design MIPs for impurities and the application of MIPs to separate impurities in APIs and drug products. Hence, we discuss the application of MIPs to separate impurities in APIs and drug products and present future perspectives related to the development of MIPs to analyse impurities.

## 2. Design of MIPs for Pharmaceutical Impurities

Molecular imprinting is used to create specific artificial recognition sites in a polymer matrix that can bind specifically and selectively to analytes (template molecules) [28,29,30]. The components involved in generating MIPs include a template molecule, a functional monomer, a crosslinker, a porogen, and an initiator [31,32,33]. The design of the MIP-synthesis process is essential to produce an end product with good analytical performance. One of the most critical factors is to determine the best functional monomer(s) that will interact selectively with template molecules. Based on the literature, researchers have identified the functional monomers used to create MIPs for impurities with and without computer simulations. In general, when researchers have selected functional monomers without computer simulation, they have used methacrylic acid (MAA) as the functional monomer [34,35,36,37,38,39,40] because it has a carboxyl group that can act simultaneously as a hydrogen donor and acceptor. This allows strong interactions between the template molecule and the monomer via hydrogen bonding [33].

Computational simulations can assist in selecting functional monomers by evaluating hydrogen interactions between template molecules and functional monomers [29]. Apart from playing a role in determining the type of functional monomer, computational simulations also play a role in determining the best monomer ratio in less time than conducting trials in the laboratory (Table 2) [41]. For example, Viveiros et al. [42] used the SYBYL™ 7.6 software for the entire computational process to identify the best functional monomer and composition for an acetamide MIP. They entered all of the tested monomer structures using Gasteiger–Hückel calculus and refined the design with molecular mechanics methods by applying energy minimization with the MAXIMIN2 command. They screened individual functional monomers of the library against the template using the LEAPFROG™ algorithm, which allows energy-based evaluation of binding values for ligand structures [42]. They ran the program for 60,000 iterations and scored the binding energies of template–monomer interactions; the highest binding score corresponds to the best combination [43]. Based on the computer simulations, they selected monomers with the highest binding score and used them in a simulated molecular dynamics or annealing process to study their interaction with acetamide as a template molecule in the presence of carbon dioxide (CO_2_) as a porogen. They also obtained the template-to-monomer ratios from this computer simulation. They selected itaconic acid with a binding score of −33.31 kcal/mol and 2-hydroxyethyl methacrylate with a binding score of −15.71 kcal/mol as the optimal monomers to interact with acetamide [42]. Based on the binding score, itaconic acid had a stronger affinity to acetamide than 2-hydroxyethyl methacrylate. Based on the experiment in the laboratory, acetamide–itaconic acid MIPs had a 2.3-fold higher affinity towards 2-hydroxyethyl methacrylate than acetamide-2-hydroxyethyl methacrylate MIPs. These results were consistent with the computational results [42].

Although MIPs can be designed without computer simulations, this approach is desirable when designing MIPs. The advantages of computational simulations are the reduction in time when determining which monomer has good binding affinity to the template molecules and better cost-effectiveness.

A computational study can be applied to select a dummy template [45], which resembles the target molecule in structure, shape, size, and function, and serves as a template for imprinting. Using a dummy template avoids template leaks that can lead to analysis errors [46]. Fu et al. [45] performed a computational study to select the dummy template for separating the GTIs. They evaluated 2,6-dichloroaniline, *p*-toluidine, and aniline as dummy templates. The template molecule and functional monomer complex were constructed to evaluate the interaction strength between aromatic amines and functional monomers at the molecular level [45]. They optimized the most stable template–monomer complex first, calculating its interaction energy, ∆E, with the equation: ∆E = E_(template−monomer)_ − E_(template)_ − E_(monomer)_ [45,47]. In this study, the authors used MAA as a functional monomer. ∆E for 2,6-dichloroaniline, *p*-toluidine, and aniline was −9.60, −8.11, and −7.88 kcal/mol, respectively. Based on the results, the authors chose aniline as a dummy template because it had the lowest binding energy with the MAA, indicating that would have the most potent effect on MAA and is the most stable compound [45].

In another study, researchers prepared theoretical MIPs, using *S*-pramipexole as a model drug and its structural analogue, S-2,6-diamino-4,5,6,7-tetrahydrobenzothiazole, as the template. The authors constructed theoretical polymeric models based on different functional monomers and ethylene glycol dimethacrylate as a crosslinker [48]. Computer modelling provides one way to study the adsorption process. This involves several steps: first, remove the template molecule from the computational model cavity. Second, insert the analyte and the solvent into the model. Third, run a computer simulation. Fourth, calculate the binding energy using the equation: ΔE_B_ = E_system_ − E_analyte_ − E_cavity_. E_system_ refers to the potential energy of the cavity with bound analyte in the solvent, E_analyte_ refers to the potential energy of the analyte, and E_cavity_ refers to the potential energy of the cavity without analyte in the solvent [48]. The authors used S-pramipexol as an analyte and included it in various MIP models to confirm that the adsorption capacity results aligned with previous experimental results [49]. The interactions between S-pramipexol in the MIP cavities constructed with itaconic acid as a functional monomer had the lowest ΔE_B_ of −114.75 kcal/mol [48]. The result correlated well with the experimental adsorption capacity [49]. The authors also conducted theoretical analysis on the selectivity of the MIP system towards a particular group of compounds, known as the S-pramipexole impurities and degradants. The template and model drug had the lowest ΔE_B_ values of −358.40 kcal/mol and −339.51 kcal/mol, respectively. However, the analysed impurities had higher ΔE_B_ values [48].

## 3. Methods Used to Synthesize MIPs for Pharmaceutical Impurities

Several synthesis methods have been developed to obtain MIPs with good performance. Based on the literature, bulk polymerization, surface polymerization, and supercritical fluid (SCF) technology have been used to synthesize MIP to separate impurities in APIs and drug preparations. In addition, particular strategies such as dummy templates have been applied to obtain MIPs. 

### 3.1. Bulk Polymerization

**Figure 2:**
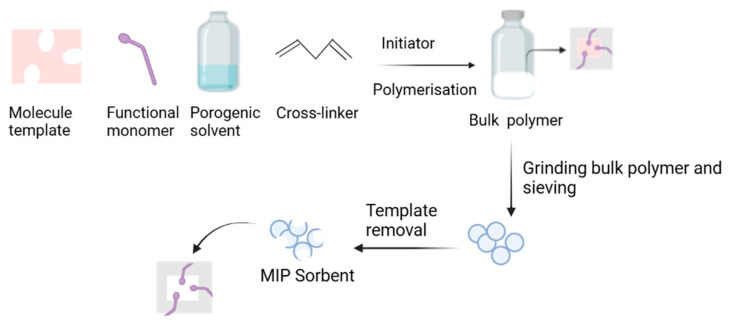
The bulk polymerization method to obtain molecularly imprinted polymers.

Bulk polymerization is a conventional method often used to prepare MIPs because it is simple and inexpensive. The template molecules, functional monomers, crosslinkers, and initiators are mixed in a porogen solvent. Polymerization is initiated by light or thermal irradiation. A block polymer is obtained with this method. Therefore, grounding, crushing, and sieving are required after polymerization (Figure 2). Table 3 lists the MIPs that have been synthesized using bulk polymerization and then applied to separate pharmaceutical impurities.

Székely et al. [50] designed MIPs using bulk polymerization to remove GTIs such as acetamide and aryl sulfonate from APIs. Acetamide is a pharmaceutical impurity that is potentially genotoxic because it can interact with DNA. Acetamide is typically present in the final stages of API manufacturing [50]. There are many sources of genotoxic arylsulfonate contamination in APIs, one of which is esterification between *p*-toluene-sulphonic acid (TsOH) and residual solvents such as methanol, which produces the genotoxic byproduct methyl tosylate (MeTs) [51]. The best-performing MIP for separating acetamide was synthesized using MAA as a functional monomer and toluene as a porogen solvent. Toluene can improve the analytical performance of MIPs because it is a nonpolar solvent that encourages polymerization by forming complexes between templates and monomers [51]. The MIP was applied as an SPE adsorbent. The authors used a mixture of acetamide and Etodolac in acetonitrile as a model sample solution (load sample). The MIP could bind 100% acetamide in the load step while the non-molecularly imprinted template (NIP) bound 77% of the acetamide [51]. The MIP for the removal of aryl sulfonate was synthesized using MeTs as the template molecule, 1-(4-vinylphenyl)-3-(3,5bis(trifluoromethyl)phenyl)urea (U) as a functional monomer, and two different crosslinkers, ethylene glycol dimethacrylate (EGDMA) or divinylbenzene, in chloroform with two ratios of T/U/EGDMA 0.1/0.1/2 (MIP 1) and 1/0.1/2 (MIP 2) or T/U/DVB 0.1/0.1/2 (MIP 3) and 1/0.1/2 (MIP 4). The authors evaluated the binding affinity of the MIPs using a solution of MeTs and halobetasol propionate (the API). MIP 1 and MIP 2 were better able to bind specifically to MeTS than the API. Compared with the NIP, MIP 1 and MIP 2 had a high imprinting factor (IF) [51].

The addition of base to the pre-polymerization solution could increase the adsorption capacity when MAA is used as a functional monomer. Székely et al. [39] added 1,2,2,6,6-pentamethylpiperidine (PMP) base to a pre-polymerization solution containing MAA as the functional monomer and 1,3-diisopropyl urea (IPU) as the template to produce MIP to remove IPU from Keppra (Kp), mometasone furoate (Meta), and roxithromycin (Roxi) as APIs. PMP converts MAA to MAA carboxylate (carboxylate anion) that can bind with two donors of NHs of IPU in a *syn* arrangement; an interaction that is stronger than neutral free acid. The MIPs were synthesized with two formulas, without PMP (MIP 1) and with PMP (MIP 2). The degree of IPU binding with MIP 2 was higher than with MIP 1 (80% and 55%, respectively) [39].

Using a dummy template is one strategy to develop a MIP. In one study, the researchers used a dummy template to avoid template leakage that could reduce the accuracy of the analysis [52]. The most commonly used dummy templates are structural analogues of the analyte [53] or isotope-labelled template [54]. Aniline was used as a dummy template to prepare a dummy MIP that could pretreat a sample containing aromatic amine GTIs. The aniline–MAA–EGDMA molar ratio of 1:4:8 produced a dummy MIP with the highest capacity to adsorb aniline (Q = 8.6 mg/g) and good blotting effect (IF = 1.3) [45]. The dummy MIP could simultaneously extract *p*-toluidine, *p*-acetotoluidide, and 2,6-dichloroaniline. The authors applied the dummy MIP as an SPE sorbent to remove 5 ppm 2,6-dichloroaniline from a diclofenac sodium sample and 10 ppm *p*-toluidine from a torasemide sample. After extraction, the solutions did not contain 2,6-dichloroaniline or *p*-toluidine, indicating that the dummy MIP could be used for quality control of the drug [45].

**Table 3:** Molecularly imprinted polymers (MIPs) that have been synthesized using the bulk polymerization method.

Sample	Impurity	Type of Impurity	Template	Binding Capacity	Imprinting Factor	Ref.
Mometasone furoate (APIs)	4-Dimethylamino pyridine	Organic (genotoxic impurity) from API post-reaction stream	4-Dimethylamino pyridine	5.03 mg/g	NM	[37]
Diclofenac sodium and torasemide	2,6-Dichloroaniline	Organic (genotoxic impurities) from synthesis, storage, or transportation of APIs	Aniline (dummy template)	4.08 mg/g	NM for 2,6-dichloroaniline or *p*-toluidine Aniline: 1.3	[45]
	*p*-Toluidine			±6 mg/g		
Keppra (Kp), mometasone furoate (Meta), and roxithromycin (Roxi) as APIs	1,3-Diisopropylurea	Organic (genotoxic impurity) from API post-reaction stream	1,3-Diisopropylurea	NM, but 80% binding for MIP synthesized when base was added	NM	[39]
Diphenhydramine hydrochloride	Benzhydrol	Organic (genotoxic impurity) from intermediate of pharmaceuticals	Benzhydrol	98.3 µmol/g	NM	[55]
Fluvoxamine maleate hydrochloride (APIs)	((2RS)-2-[[2-[[[(1E)-5-methoxy- 1-[4(trifluoromethyl) phenyl] pentylidene]amino] oxy]ethyl]amino] butanedioic acid	Organic	((2RS)-2-[[2-[[[(1E)-5-methoxy- 1-[4(trifluoromethyl) phenyl] pentylidene]amino] oxy]ethyl]amino] butanedioic acid	100 µg/mg	NM	[34]

NM, not mentioned in the article.

### 3.2. Surface-Imprinting Polymerization

**Figure 3:**
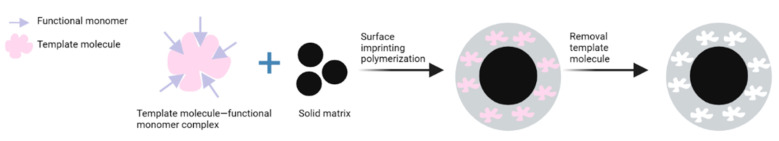
The surface molecular imprinting process.

Surface-imprinting polymerization has been developed to overcome the drawbacks of the conventional bulk and precipitation MIP-synthesis methods [56]. The imprinted materials were thick and needed large amounts of solvent to remove the molecule template [57]. In this method, molecule imprinting occurs on the surface of the solid matrix where recognition sites are distributed on the layer. The advantages of this method are the ability to reduce ‘embedding’ incidents, to promote efficient mass transfer, and to enhance the adsorption capacity [58]. The solid matrix commonly used in this method is silica nanoparticles and Fe_3_O_4_ (the magnetic component). In general, surface-imprinting polymerization occurs via three steps: (i) synthesis of the solid matrix, (ii) modification of the solid matrix, and (iii) surface molecular imprinting. The latter step begins by forming a monomer–template molecular complex under certain conditions. Then, polymerization occurs on the surface of the solid matrix with an initiator and crosslinker. After polymerization, the template molecules are removed to form cavities identical to those of the template molecule (Figure 3) [58]. Several studies have been carried out to synthesize MIPs to separate impurities using the surface polymerization method (Table 4).

Hashemi-Moghaddam and Abbasi [35] synthesized a surface molecularly imprinted on silica nanoparticles to remove the *p*-nitrophenol (4-NP) from paracetamol. 4-NP is used as an intermediate in manufacturing pharmaceuticals such as analgesics/antipyretics It is an impurity in medicinal substances that causes carcinogenic risks to humans [59]. Silica nanoparticles were obtained by hydrolysis of tetraethyl orthosilicate (TEOS); it was functionalized using 3-(methacryloxy)propyltrimethoxysilane (MPTS) to obtain the vinyl end groups. MAA was used as a functional monomer for surface molecular imprinting. The maximum adsorption capacity was 72 × 10^−3^ mmol/L. The MIP (5 mg) could adsorb 85% of 4-NP at 10 ppm, while 5 mg of the NIP adsorbed 4% of 4-NP at the same concentration. These findings indicate that the MIP has a recognition site that provides better adsorption than the NIP. The selectivity factor is a ratio of the distribution factor of the analyte (molecule template) with a similar compound. In this study, the selectivity factor of the MIP between 4-NP and paracetamol was 18.48. Meanwhile, the selectivity factor of NIP was 2.66. Hence, the MIP selectively bound 4-NP [35].

A magnetic component (Fe_3_O_4_) is a popular solid matrix used to synthesize MIP. The MIPs synthesized using a solid magnetic matrix are called magnetic molecularly imprinted polymers (MMIPs). These MMIPs can be directly applied to the sample solution, and an external magnet can be used for the separation process [60]. MMIPs for the removal of sulphanilamide has been synthesized using Fe_3_O_4_ as a solid magnetic matrix. Then, the surface of Fe_3_O_4_ was functionalized with SiO_2_ and 3-methacryloxypropyl trimethoxy-silane (MPTS) (Fe_3_O_4_@SiO_2_@MPTS). Sulphanilamide is a major degradation product of sulphacetamide preparations; it is formed when exposed to light, extreme temperatures, or long storage [61,62]. In one study, researchers used sulphanilamide as a template molecule, MAA as a functional monomer, EGDMA as a crosslinker, and 2,2-azobisisobutyronitrile (AIBN) as a radical initiator in a mixture of acetonitrile and toluene (60:40, *v*/*v*) as a porogen [36]. The adsorption capacity of the MMIP was 114.2 µmol/g. The authors applied it to separate sulphanilamide after spiking 10 mL of eye drop solution with 10 mL of 0.1 mmol^−1^ sulphanilamide, then diluting the solution to 50 mL with water. They adjusted the pH to 6.0 and then mixed 10 mL of this solution with 0.1 g of the MMIP for 30 min. They injected the supernatant into a high-performance liquid chromatography column. The sulphanilamide peak intensity decreased after purification while the sulfacetamide (the API) peak intensity did not decrease, indicating that the synthesized MMIP had good selectivity [36].

Luo et al. [38] confirmed that surface polymerization could overcome the drawback of bulk polymerization. They synthesized a MIP with surface polymerization (S-MIP) using SiO_2_ modified by 3-aminopropyl triethoxysilane (SiO_2_-APTES) as a solid matrix. They used penicilloic acid as a template, MAA as a functional monomer, and EGDMA as a crosslinker. They also synthesized a MIP with bulk polymerization (B-MIP) using the same conditions but without a solid matrix (SiO_2_-APTES) [38]. Based on the adsorption isotherm, the saturated adsorption capacity of S-MIP was 22.67 mg/g; much higher than the B-MIP (10.31 mg/g). The IFs for S-MIP and B-MIP were 6.3 and 2.2, respectively. The S-MIP showed better analytical performance than the B-MIP [38]. The S-MIP reached adsorption equilibrium (45 min) faster than B-MIP (90 min). The template required longer to reach adsorption equilibrium with the B-MIP due to embedded active sites [38]. In the S-MIP, most of the template binding sites were located on the surface of the polymer, enhancing the molecular recognition ability between the polymer and the target compound and improving the mass transfer kinetics of the S-MIP [63]. 

### 3.3. SCF Technology

Based on environmental regulations and safety hazards, the pharmaceutical industry is trying to reduce the use of organic solvents [64]. SCFs are one alternative to replace hazardous organic solvents with environmentally friendly approaches. SCFs are highly compressible gases: they exceed a liquid’s critical temperature and pressure but are below the pressure required to condense from the liquid to the solid state [65]. SCFs have many advantages as green solvents, such as being non-toxic, chemically inert, non-flammable, of a high purity, low-cost, and easily removed [66]. In addition, SCFs have been applied as a solvent for molecularly imprinted technologies because they can dissolve the majority of monomers [44].

**Figure 4:**
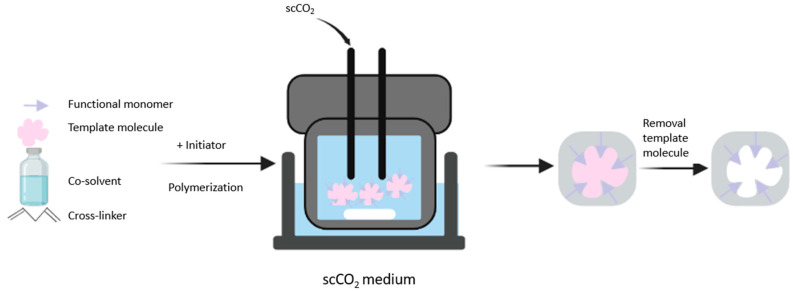
Schematic of the synthesis of molecularly imprinted polymers using supercritical carbon dioxide fluid.

Polymerization is carried out in a stainless steel high-pressure reactor. In one study, the authors introduced the template molecule, functional monomer, crosslinker, and initiator into the reactor immersed in a thermostat water bath. They added CO_2_ up to 21 MPa and performed polymerization for 24 h with stirring. The next step was desorption of the template molecule using supercritical CO_2_ or a co-solvent to obtain the specific binding sites [44]. Figure 4 illustrates the synthesis of MIPs using supercritical CO_2_. The studies that have applied supercritical CO_2_ to synthesize MIPs are summarized in Table 5. 

Viveiros et al. [44] synthesized MIPs to purify acetamide from an API. The authors used MAA and methacrylamide (MAM) as functional monomers. They performed four different polymerizations: (i) MAA with 0.5 mL acetonitrile as a co-solvent (MIP 1), (ii) MAA without a co-solvent (MIP 2), (iii) MAM with 0.5 mL acetonitrile as a co-solvent (MIP 3), and (iv) MAM without a co-solvent (MIP 4). Based on the static binding analysis, MIP 3 had a higher adsorption ability of acetamide at 250 ppm and an IF of 1.31. The adsorption capacity (Qmax) based on the Langmuir isotherm of MIP 3 was 2.99 mmol/g. The MAM and acetamide interactions were stronger than the MAA and acetamide interactions. The structural similarities between MAM and the acetamide also lead to a higher affinity for it than for MAA [44]. 

Viveiros et al. [44] synthesized two kinds of MIPs for selective removal of acetamide in APIs using benzamide (BENZ) and pivalamide (PIV) as dummy templates (MIP 1 and MIP 2, respectively). BENZ is a planar-shaped template molecule, while PIV is a three-dimensional analogue template molecule. They used supercritical CO_2_ for the synthesis. MIP 1 and MIP 2 were free-flowing, dry, ready-to-use, and homogenous powders. The advantages of using supercritical CO_2_ compared with an organic solvent were the absence of residual solvent and insufficient grinding and sieving [63]. In addition, MIP 2 showed a higher adsorption capacity of all amide-based compounds in the static binding study (acetamide, BNZ, and PIV) than MIP 1. This is because MIP 2 has a three-dimensional cavity that is more accessible than MIP 1 (which has a planar cavity). In a dynamic study using an mixed solution (acetamide, BNZ, and PIV), MIP 2 could remove 32% more acetamide than MIP 1, making it potentially applicable for the removal of amide-based genotoxins from crude pharmaceutical mixtures [67].

Based on Table 3, Table 4 and Table 5, organic compounds have commonly been used to develop MIPs that recognize pharmaceutical impurities. These impurities are usually generated during API synthesis or are due to product degradation. However, MIPs for impurities have not been developed for other types of impurities such as heavy metals, inorganic salts, reagents, and residual solvents. Ionic MIPs could be developed to separate heavy metal impurities in APIs. There may be problems with the development of MIPs for these impurities because an API could contain more than one type of impurity, so the development of these MIPs could take more time. This potential disadvantage could be overcome by using multi-template molecularly imprinted polymers (MT-MIPs). This simple and reliable approach can be used to efficiently remove and enrich multiple analytes simultaneously in a single process [68,69]. In addition, factors that can become obstacles in the MIPs development process are related to impurity raw materials used as templates, such as unavailable, toxic, expensive, and unstable raw materials for the synthesis process. This problem might be overcome by using a dummy template, as Fu et al. [45] did.

The advantages and disadvantages of the polymerization methods used to synthesize MIPs to separate impurities are listed in Table 6. 

## 4. Conclusions

MIPs can be used to separate impurities in APIs and drug products. In addition, they can be applied in the preparation process to determine the levels of impurities in APIs or drug products. Computer simulations are a good choice to guide the selection of functional monomers and to determine the template-to-monomer ratio. This approach produces MIPs with better performance in a shorter amount of time. The most common methods used in MIP synthesis for impurities are bulk polymerization, surface-imprinting polymerization, and SCF technology. Based on the comparison of the bulk and surface-imprinting polymerization, the MIPs produced by the latter method have a higher adsorption capacity and faster adsorption. Overall, the application of MIPs to analyse APIs and drug products as well as adsorbents for purification, is still relatively low, considering that MIPs have the advantage of separating impurities to increase separation efficiency selectively. Future research involving the use of MIPs to separate and analyse impurities in pharmaceutical products should focus on the following:

1. Develop MIPs for other types of impurities. Ionic MIPs can be developed to detect and separate heavy metals in pharmaceutical products.
2. Compare the analytical performance of MIPs obtained using SCF technology with those obtained using other methods. In addition, compare the costs required for each technique to determine cost-effectiveness and analytical performance.
3. Develop MT-MIPs to separate multiple impurities simultaneously and to reduce the time required for analysis.

## Figures and Tables

**Table 1:** The methods that have been employed to analyse impurities in active pharmaceutical ingredients (APIs) and drug products.

Sample	Impurity	Preparation Method	Instrument	Accuracy	Precision	Ref
API Crotamiton	Toluidine	Dissolved in methanol	Gas chromatography with flame ionization detector (GC-FID)	79.1–107.4%	2.1–4.3%	[12]
APIs of various sartans containing a tetrazole group	4′-(Azidomethyl)-[1,1′-biphenyl]-2-carbonitrile (GTI-azide-1) and 5-(4′-(azidomethyl)-[1,1′-biphenyl]- 2-yl)-1H-tetrazole (GTI-azide-2)	Dissolved and sonicated	High-performance liquid chromatography coupled with mass spectrometry (HPLC-MS)	GTI-azide-1: 100.9% GTI-azide-2: 100.4%	GTI-azide-1: 0.25% GTI-azide-2: 1.39%	[13]
4,4′-(propanediamido)dibenzoate (malaben)	Impurities A (4-aminobenzoic acid), B (unidentified), C (Etmaben), and D (unidentified)	Dissolved in water	Capillary electrophoresis	NM	NM	[14]
APIs and market-authorized tablets	*N*-nitrosamines	Extraction, removal using cation exchange resin, enrichment using charcoal, and evaporation	Liquid chromatography–tandem mass spectrometry (LC-MS/MS)	83.8–113.3%	0.9–14.9%	[15]
Rifampicin capsule	4-Methyl-1-nitrosopiperazine	Dissolved and vortexed	LC-MS/HRMS	NM	NM	[16]
Metronidazole APIs	Cd, Pb, As, Hg, Co, Ni, Ag, Cu, Sn, and Cr	Heated at 250 °C	Inductively coupled plasma optical emission spectrometry	NM	<2%	[17]
Drug substances of sartans, metformin, ranitidine, and their finished products	*N*-Nitrosodimethylamine (NDMA) and *N*-nitrosodiethylamine (NDEA)	Precipitation using the solubility difference method for irbesartan, pimasartan, olmesartan, and candesartan samples Solid-phase extraction with activated charcoal for valsartan, rosartan, metformin, and ranitidine samples	Gas chromatography–tandem mass spectrometry	NMDA: 95.0–105% NDEA: 93.6–104%	NMDA: 0.4–2.7% NDEA: 0.4–4.2%	[18]
Ranitidine dosage forms	NDMA	Ultrasonic extraction	Electrospray ionization–liquid chromatography–tandem mass spectrometry (ESI-LC-MS/MS)	94.7–102.0%	4.9%	[19]

NM, not mentioned in the article.

**Table 2:** Comparison of static binding capacities in standard acetamide solutions (250 ppm) for molecularly imprinted templates designed with and without computer simulations.

Design	Template (T)	Monomer (M)	Ratio of T:M	Static Binding Capacity (mmol/g)	Ref.
With computer simulations	Acetamide	Itaconic acid	1:3	±2.5	[42]
		2-Hydroxyethyl methacrylate	1:2	±1.1	
Without computer simulations		Methacrylic acid	1:4	±1.7	[44]
		Methacrylamide	1:4	±2.3	

**Table 4:** Molecularly imprinted polymers (MIPs) that have been synthesized using surface polymerization.

Sample	Impurity	Type of Impurity	Solid Matrix	Template	Monomer	Porogen	Binding Capacity	Selectivity Factor	Ref.
Paracetamol	*p*-Nitrophenol (4-NP)	Organic from an intermediate of pharmaceuticals	Silica nanoparticle	*p*-Nitrophenol	Methacrylic acid	Toluene–acetonitrile (4:1, *v*/*v*)	600 mol/g	18.48	[35]
Sulphacetamide eye drops	Sulphanilamide	Organic from a degradation product	Fe_3_O_4_@SiO_2_ @MPTS	Sulphanilamide	Methacrylic acid	acetonitrile/toluene (60:40, *v*/*v*)	114.2 µmol/g	NM	[36]
Penicillin	Penicilloic acid	Organic (genotoxic impurities) from a degradation product	SiO_2_ modified by 3-aminopropyl triethoxysilane (APTES)	Penicilloic acid	Methacrylic acid	Acetonitrile/ methanol (1:1)	22.67 mg/g	NM, but the IF of penicilloic acid was higher than other compounds	[38]

NM, Not mentioned.

**Table 5:** Synthesis of molecularly imprinted polymers using supercritical carbon dioxide as a solvent.

Special Strategies	Impurity	Type of Impurity	Template	Monomer	Solvent	Static Binding Capacity	Imprinting Factor	Selectivity Factor	Ref.
-	Acetamide	Organic from the last stages of API manufacturing	Acetamide	Methacrylamide	Supercritical CO_2_ and acetonitrile (co-solvent)	±2.3 mmol/g (at 250 ppm)	1.31 (at 250 ppm)	NM, but the MIP had higher affinity for acetamide than either benzamide or pivalamide	[44]
-	Acetamide	Organic from the last stages of API manufacturing	Acetamide	Itaconic acid	Supercritical CO_2_	2.5 mmol/g	NM	NM, but the MIP had higher affinity for acetamide than either benzamide or pivalamide	[42]
Dummy template	Acetamide	Organic from the last stages of API manufacturing	Benzamide	Methacrylic acid	Supercritical CO_2_	1.26 mmol/g for acetamide	2.04	NM	[67]
	Acetamide	Organic from the last stages of API manufacturing	Pivalamide	Methacrylic acid	Supercritical CO_2_	1.33 mmol/g for acetamide	0.88		

NM, Not mentioned.

**Table 6:** The advantages and disadvantages of the polymerization methods used to synthesize molecularly imprinted polymers (MIPs) to separate impurities.

Polymerization Method	Advantages	Disadvantages
Bulk polymerization	Easy procedure Requires a small amount of porogen	Grinding involved in this method can damage the recognition site of MIPs MIPs are irregularly shaped
Surface-imprinting polymerization	Can increase the binding capacity Improves the mass transfer kinetics Faster adsorption equilibrium	Quite complicated because it involves many steps
Supercritical fluid technology	Uses a green and highly pure solvent The MIPs are obtained as dry free-flowing powder The MIPs are ready to use	Requires special equipment for polymerization

## Data Availability

Data sharing is not applicable.
